# Datasets for structural and mechanical properties of nanoporous networks from FIB reconstruction

**DOI:** 10.1016/j.dib.2025.112152

**Published:** 2025-10-08

**Authors:** Yong Li, Kaixiong Hu, Erica T. Lilleodden, Norbert Huber

**Affiliations:** a Institute of Hydrogen Technology, Hybrid Materials Systems, Helmholtz-Zentrum Hereon, Geesthacht, Germany; b School of Transportation and Logistics Engineering, Wuhan University of Technology, Hubei, China; c Institute for Microstructure of Materials and Systems IMWS, Halle, Saale, Germany; d Bundesanstalt für Materialforschung und -prüfung, Berlin, Germany; e Institute of Materials Physics and Technology, Hamburg University of Technology, Hamburg, Germany

**Keywords:** Nanoporous gold, Dealloying, FIB/SEM tomograph, Finite element, Volume mesh, Young’s modulus, Yield stress, Poisson’s ratio

## Abstract

This dataset paper presents a comprehensive archive of 3D tomographic reconstruction image files, volume mesh files for finite element simulations, and tabulated structural and mechanical properties data of nanoporous gold structures. The base material is nanoporous gold, fabricated using a dealloying process, with a solid fraction of approximately 0.30. The NPG samples with ligament sizes ranging from 20 nm to 400 nm were prepared by dealloying and by controlling the thermal annealing process. The original data consist of tomographic TIFF files acquired through Focused Ion Beam/Scanning Electron Microscopy (FIB/SEM) 3D reconstruction, as detailed in Philosophical Magazine 2016 96 (32-34), 3322-3335. At each ligament size, six sets of 3D tomographic images were obtained from different regions of the same sample to ensure representative data. New simulations and analyses were conducted based on the 3D image data. The resulting structural and mechanical property data of nanoporous gold are reported for the first time in this dataset paper. Volume meshing of the 3D reconstructed data was performed using Simpleware software. Structural parameters, including surface area, solid volume, and solid volume fraction of the nanoporous network, were extracted from the meshed volumes. Structural connectivity was assessed from the 3D microstructures. The meshed volumes were then used as input for finite element simulations performed in Abaqus to evaluate mechanical responses under uniaxial compression along all three principal axes respectively. From the resulting stress–strain curves, the Young’s modulus and yield strength of each structure were determined. Both elastic and plastic Poisson’s ratios were analyzed from true strain increments. This dataset includes the 3D tomographic images, corresponding volume mesh files, mechanical behavior data and tables summarizing the structural and mechanical properties. The archived data serve as a database for nanoporous network materials and can be reused for numerical simulations, additive manufacturing, and machine learning applications within the materials science community. All files are openly accessible via the TORE repository at https://doi.org/10.15480/882.15230.

Specifications Table

**Table:** 

Subject	Materials Science
Specific subject area	FIB/SEM tomographic reconstructions and volume meshes of nanoporous gold networks
Type of data	Raw and analysed data: 1.TIFF image files from FIB (Focused Ion Beam)/SEM (Scanning Electron Microscopy) tomographic reconstructions of nanoporous gold with varying ligament size 2. Abaqus input files containing volume mesh data derived from the tomographic images 3. Engineering stress-strain data of uniaxial compressive loading in all three principal axes 4. Poisson’s ratio data during compression 5. Tables summarizing the structural and mechanical properties of each nanoporous gold structure
Data collection	The experimental procedures for sample preparation and for FIB/SEM tomography are detailed in Ref. [1], while the numerical simulations and post-processing analyses are described within this dataset. 1. The 3D nanoporous structure was reconstructed using FIB/SEM tomography. 2. Volume meshing was performed using Simpleware (Synopsys, Inc.). 3. Mechanical properties were evaluated using the finite element method in Abaqus. 4. Structural connectivity was calculated using the open-source software CHomP.
Data source location	Hamburg University of Technology, open-source repository: TORE
Data accessibility	Repository name: TORE Data identification number: https://doi.org/10.15480/882.15230 Direct URL to data: https://hdl.handle.net/11420/55753
Related research article	Kaixiong Hu, Markus Ziehmer, Ke Wang and Erica T. Lilleodden Nanoporous gold: 3D structural analyses of representative volumes and their implications on scaling relations of mechanical behaviour Philosophical Magazine 2016 96 (32–34), 3322–3335 https://doi.org/10.1080/14786435.2016.1222087

## 1 Value of the Data

• The 3D tomographic nanoporous network structures are the reconstruction of experiments with nanoporous gold samples prepared with various ligament size range from 20 nm to 400 nm. They may be used for further study of process-structure-property relationships of nanoscale networks.
• The volume meshing of nanoporous network can directly be reused for numerical simulations, such as finite-element modelling, for the research on the mechanical properties and functionalities on the nanoporous structure, such as the thermal and electrical conductivity, or the diffusion properties of the nano-size pores.
• The tomography image data and the volume mesh data can also be reused as the input configuration for the additive manufacturing to prepare the porous structure for wide range of materials such as ceramics, polymers and metals with arbitrary length scale.
• The simulation models derived from experiments serve for validation of computational approaches that are needed for generating big datasets of microstructures and mechanical properties. These can be used as input for training and validation of machine-learning models of nanoporous materials.

## 2 Background

The datasets on numeric nanoporous gold (NPG) structures generated by the leveled-wave (LW) method [2] and the coarsened structures obtained through kinetic Monte Carlo simulations [3] have been archived by Li et al. in Ref. [4]. The numeric NPG microstructure dataset combined with FE simulations offered the opportunity to establish the link between microstructure, topology and mechanical properties of NPG [5]. However, corresponding experimental datasets remain scarce which limits the validation of the prediction. The dataset presented here, along with the archived data, represents an important step toward closing this gap.

According to Richert & Huber, the statistical descriptors of ligament cross-sections evolve from LW NPG structures to coarsened NPG structures, where their distributions approach those of FIB/SEM 3D reconstruction tomographs[6]. While the cross-sections of initial LW-generated microstructures often contain unphysical, non-round shapes, this can systematically bias the results and lead to an overprediction of Young’s modulus. This bias is particularly relevant for the validation of convolutional neural networks (CNNs) trained to predict the mechanical properties of nanoporous metals from LW-based microstructure data. Robust validation requires testing the generalization capabilities of such CNNs on (a) coarsened microstructures and (b) real FIB/SEM experimental data, as proposed by Chen-Wiegart et al. [7]. Otherwise, a CNN trained solely on LW-generated cross-sections will tend to interpret any new structure according to the structure–property relationships embedded in the training data, thereby limiting its predictive reliability.

## 3 Data Description

**Fig. 1:**
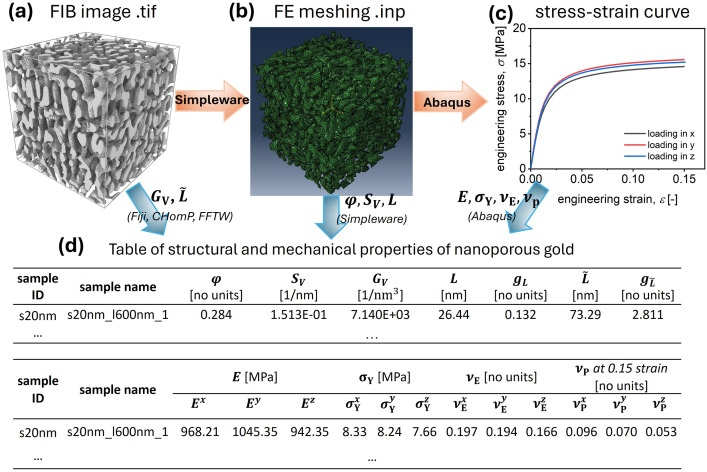
Workflow of the dataset. **(a)** The 3D tomographic reconstruction obtained via FIB/SEM imaging reveals a bi-continuous nanoporous gold network. **(b)** The corresponding volume mesh, generated using Simpleware, depicts the tetrahedral elements (C3D10). **(c)** Engineering stress–strain curves computed with Abaqus shows the mechanical response of the samples. **(d)** A summary table lists the structural and mechanical parameters derived from the previous steps. The listed parameters are defined in Table 2.

This dataset comprises four primary types of data: 1) 3D tomographic reconstruction image files of experimentally prepared nanoporous gold (NPG) structures, 2) volume mesh files generated from the tomography data, 3) mechanical response data, including stress-strain behaviour and Poisson’s ratio, obtained from finite element simulations, and 4) tables listing the structural and mechanical properties of the NPG samples. Fig. 1 outlines the overall workflow. The 3D tomographic images, reconstructed via FIB/SEM from NPG samples fabricated through dealloying or post-annealing, reveal a bi-continuous nanoporous network (Fig. 1a). Five different nanoporous structures were included, each characterized by a distinct ligament size L (the diameter of the nanoscale branches) ranging from 20 nm to 400 nm (see Table 1). The sample with *L* = 20 nm represents the as-dealloyed state, while the other samples with larger ligament sizes were obtained through surface diffusion during thermal annealing. The 3D tomographic images were then segmented to generate volume meshes using Simpleware (Synopsys, Inc.) (Fig. 1b). The resulting meshes are composed of quadratic tetrahedral elements (C3D10) that conform to the geometry of the nanoporous structure. This volume meshes were used as input for uniaxial compressive mechanical simulations conducted using the finite element software Abaqus (Fig. 1c). Based on the simulations and data analyses, key structural parameters−such as solid fraction ($\varphi_{0}$ and $\varphi_{m}$), Genus density ($G_{V}$), specific surface area ($S_{V}$), ligament size (L and $\tilde{L}$), and scaled genus (a measure of connectivity) ($g_{L}$ and $g_{\tilde{L}}$), as well as mechanical properties−including Young’s modulus (E), yield stress ($\sigma_{Y}$), Poisson’s ratio ($\nu_{E}$ and $\nu_{P}$) were evaluated and tabulated for each sample (Fig. 1d).

**Table 1:** Parameters of 3D tomographic images. Mean ligament (diameter) size, the number of voxels, voxel size and sample size of the 3D tomographic reconstruction images.

sample ID	direction	s20nm	s50nm	s200nm	s350nm	s400nm
Mean ligament size [nm]		26	47	212	366	425
No. of voxels	x	168	200	288	240	448
	y	168	200	288	240	448
	z	200	200	360	300	348
voxel size [nm]	x	3.57	5.00	12.50	25.00	17.86
	y	3.57	5.00	12.50	25.00	17.86
	z	3.00	5.00	10.00	20.00	23.00
sample size [nm]	x	600	1000	3600	6000	8000
	y	600	1000	3600	6000	8000
	z	600	1000	3600	6000	8000

Table 1 provides details of the 3D tomographic image data, including mean ligament diameter size, the number of voxels, voxel dimensions, and the total sample size. The voxel thickness in the *z*-(slicing) direction is comparable to that in the x and y directions (cross-section), ensuring near-isotropic voxel. The voxel resolution was tailored to match the characteristic structural scale of each sample and increases with ligament size. The representative volume (RV) is defined as the minimum volume necessary to accurately capture key microstructural parameters and mechanical properties of the NPG sample material. According to Ref. [8], the edge length of the RV should be >15 times the ligament size. All sample volumes included in this dataset exceed the minimum RV sizes, ensuring statistical representativeness in the structural and mechanical analyses.

Table 2 presents the dataset of structural and mechanical parameters for nanoporous gold (NPG) samples with varying ligament sizes. The listed parameters include solid volume fraction, ($\varphi_{0}$-before and $\varphi_{m}$-after the removal of isolated clusters), specific (per solid volume) surface area, $S_{V\;}$, genus density in total volume, $G_{V}$, ligament size, (L-ligament diameter, Eq.6, and $\tilde{L}$-ligament spacing, Eq.7), scaled genus, ($g_{L}$, Eq.4, and $g_{\tilde{L}}$, Eq.5), Young’s modulus, E, yield stress, $\sigma_{Y}$, elastic Poisson’s ratio $\nu_{E}$, and plastic Poisson’s ratio, $\nu_{p}$. The values of E, $\sigma_{Y}$, $\nu_{E}$ and $\nu_{p}$ for uniaxial compressive loading in all the x, y, z directions were calculated and listed.

**Table 2:** Structural and mechanical properties of NPG samples at different ligament sizes. Solid fraction, $\varphi_{0}$-before and $\varphi_{m}$-after the removal of isolated clusters. Specific (per solid volume) surface area, $S_{V}$; Genus density in total volume, $G_{V};$ The two types of ligament size: ligament diameter, L (Eq.6) and ligament spacing, $\tilde{L}$ (Eq.7); The corresponding scaled genus, $g_{L}$ (Eq.4) and $g_{\tilde{L}}$ (Eq.5). Young’s modulus, E, yield stress, $\sigma_{Y}$, elastic ($\nu_{E}$) and plastic ($\nu_{P}$) Poisson’s ratio. The values of E, $\sigma_{Y}$, $\nu_{E}$ and $\nu_{p}$ of sample under uniaxial compressive loading in the x, y, z directions were evaluated and listed. For $\sigma_{Y}$, the stress at 0.2 % ($\sigma_{Y,0.2\%}$) and 2 % ($\sigma_{Y,2\%}$) plastic strain were listed. For $\nu_{P}$, the Poisson’s ratio at a strain of 0.15 was listed.

sample ID	sample name	$\varphi_{0}$ [no units]	$\varphi_{m}$ [no units]	$S_{V}$ [1/nm]	$G_{V}$ [1/$nm^{3}$]	L [nm]	$g_{L}$ [no units]	$\tilde{L}$ [nm]	$g_{\tilde{L}}$ [no units]
**s20nm**	s20nm_l600nm_1	0.300	0.284	1.513E-01	7.140E-06	26.44	0.132	73.29	2.811
	s20nm_l600nm_2	0.298	0.284	1.512E-01	7.376E-06	26.46	0.137	73.17	2.890
	s20nm_l600nm_3	0.291	0.275	1.541E-01	7.254E-06	25.95	0.127	73.14	2.838
	s20nm_l600nm_4	0.290	0.274	1.527E-01	6.742E-06	26.19	0.121	73.62	2.690
	s20nm_l600nm_5	0.293	0.278	1.530E-01	6.952E-06	26.14	0.124	74.38	2.860
	s20nm_l600nm_6	0.291	0.276	1.524E-01	6.774E-06	26.24	0.122	71.96	2.524
**s50nm**	s50nm_l1000nm_1	0.299	0.283	8.548E-02	1.059E-06	46.79	0.108	115.45	1.629
	s50nm_l1000nm_2	0.305	0.291	8.404E-02	1.080E-06	47.60	0.116	115.83	1.678
	s50nm_l1000nm_3	0.296	0.281	8.588E-02	1.047E-06	46.58	0.106	116.02	1.635
	s50nm_l1000nm_4	0.302	0.286	8.438E-02	1.019E-06	47.40	0.109	116.18	1.599
	s50nm_l1000nm_5	0.292	0.276	8.648E-02	1.030E-06	46.25	0.102	122.69	1.902
	s50nm_l1000nm_6	0.296	0.282	8.639E-02	1.023E-06	46.30	0.102	116.51	1.618
**s200nm**	s200nm_l3600nm_1	0.321	0.306	1.799E-02	7.978E-09	222.36	0.088	606.62	1.781
	s200nm_l3600nm_2	0.317	0.305	1.836E-02	7.487E-09	217.90	0.077	518.68	1.045
	s200nm_l3600nm_3	0.302	0.287	1.854E-02	5.823E-09	215.76	0.058	532.62	0.880
	s200nm_l3600nm_4	0.290	0.262	1.911E-02	5.626E-09	209.33	0.052	519.03	0.787
	s200nm_l3600nm_5	0.279	0.261	1.944E-02	5.119E-09	205.80	0.045	541.58	0.813
	s200nm_l3600nm_6	0.273	0.246	1.975E-02	4.954E-09	202.50	0.041	560.02	0.870
**s350nm**	s350nm_l6000m_1	0.360	0.343	1.115E-02	2.212E-09	358.74	0.102	912.43	1.680
	s350nm_l6000m_2	0.363	0.347	1.081E-02	2.621E-09	370.04	0.133	956.47	2.293
	s350nm_l6000m_3	0.352	0.334	1.092E-02	2.050E-09	366.38	0.101	861.57	1.311
	s350nm_l6000m_4	0.351	0.335	1.095E-02	2.272E-09	365.20	0.111	910.28	1.713
	s350nm_l6000m_5	0.353	0.340	1.099E-02	2.192E-09	363.96	0.106	890.36	1.547
	s350nm_l6000m_6	0.349	0.328	1.081E-02	1.933E-09	370.08	0.098	887.29	1.350
**s400nm**	s400nm_l8000nm_1	0.337	0.328	9.415E-03	1.261E-09	424.87	0.097	997.20	1.251
	s400nm_l8000nm_2	0.339	0.329	9.458E-03	1.241E-09	422.91	0.094	1019.57	1.315
	s400nm_l8000nm_3	0.334	0.323	9.346E-03	1.084E-09	427.98	0.085	943.63	0.911
	s400nm_l8000nm_4	0.332	0.319	9.375E-03	1.105E-09	426.67	0.086	977.03	1.030
	s400nm_l8000nm_5	0.338	0.323	9.382E-03	1.193E-09	426.34	0.092	937.90	0.984
	s400nm_l8000nm_6	0.336	0.326	9.436E-03	1.181E-09	423.92	0.090	1014.21	1.233

sample ID	sample name	E [MPa]			$\sigma_{Y,0.2\%}$ [MPa]			$\sigma_{Y,2\%}$ [MPa]			$\nu_{E}$ [no units]			$\nu_{p,0.15}$ [no units]		
		$E^{x}$	$E^{y}$	$E^{z}$	$\sigma_{Y,0.2\%}^{x}$	$\sigma_{Y,0.2\%}^{y}$	$\sigma_{Y,0.2\%}^{z}$	$\sigma_{Y,2\%}^{x}$	$\sigma_{Y,2\%}^{y}$	$\sigma_{Y,2\%}^{z}$	$\nu_{E}^{x}$	$\nu_{E}^{y}$	$\nu_{E}^{z}$	$\nu_{p}^{x}$	$\nu_{p}^{y}$	$\nu_{p}^{z}$
**s20nm**	s20nm_l600nm_1	968.2	1045.4	942.4	8.33	8.24	7.66	13.42	13.12	12.20	0.197	0.194	0.166	0.084	0.050	0.032
	s20nm_l600nm_2	1080.6	1133.7	1053.2	9.01	9.18	8.23	14.46	14.38	13.26	0.172	0.191	0.176	0.098	0.076	0.051
	s20nm_l600nm_3	938.3	1087.1	935.9	8.01	8.73	8.14	12.85	13.90	12.82	0.171	0.207	0.156	0.057	0.092	0.061
	s20nm_l600nm_4	918.8	1038.2	943.2	7.39	7.81	8.14	12.04	12.87	12.71	0.175	0.200	0.172	0.073	0.074	0.059
	s20nm_l600nm_5	846.9	841.7	850.2	7.03	7.07	7.40	11.72	11.58	11.76	0.186	0.180	0.169	0.054	0.061	0.052
	s20nm_l600nm_6	959.2	918.4	873.9	8.24	7.81	7.50	13.04	12.77	12.13	0.196	0.210	0.190	0.098	0.096	0.063
**s50nm**	s50nm_l1000nm_1	605.7	858.8	1376.0	5.22	6.94	10.07	9.02	11.89	16.17	0.138	0.205	0.214	0.046	0.076	0.064
	s50nm_l1000nm_2	656.7	1117.7	1376.0	6.11	9.57	11.88	10.55	15.25	17.48	0.120	0.182	0.206	0.045	0.087	0.075
	s50nm_l1000nm_3	518.6	740.9	1168.3	5.09	5.74	8.28	8.80	9.84	13.29	0.120	0.153	0.299	0.045	0.050	0.102
	s50nm_l1000nm_4	512.2	889.0	1339.8	4.65	7.47	9.80	8.05	12.48	15.31	0.125	0.186	0.224	0.071	0.096	0.049
	s50nm_l1000nm_5	513.3	793.8	1266.3	4.96	7.06	9.14	8.51	11.76	14.48	0.114	0.165	0.252	0.043	0.072	0.080
	s50nm_l1000nm_6	535.0	821.6	1113.8	5.15	7.04	8.41	8.73	11.70	13.36	0.126	0.174	0.204	0.039	0.092	0.046
**s200nm**	s200nm_l3600nm_1	436.7	723.4	213.7	3.82	5.68	2.10	6.65	9.33	3.83	0.162	0.191	0.072	0.047	0.085	0.025
	s200nm_l3600nm_2	281.3	518.3	161.8	2.82	4.28	1.71	4.72	6.98	3.35	0.184	0.279	0.103	0.064	0.141	0.063
	s200nm_l3600nm_3	106.1	227.8	66.3	1.12	2.36	1.02	1.85	3.97	1.77	0.161	0.325	0.061	0.106	0.255	0.051
	s200nm_l3600nm_4	140.1	188.0	43.8	1.30	1.80	0.62	2.26	3.10	1.05	0.202	0.211	0.054	0.050	0.032	0.021
	s200nm_l3600nm_5	126.4	179.0	57.2	1.24	1.71	0.76	2.41	2.73	1.24	0.215	0.218	0.106	0.094	0.069	0.034
	s200nm_l3600nm_6	71.0	136.5	34.6	0.80	1.47	0.55	1.38	2.50	0.97	0.082	0.414	0.053	0.078	0.234	0.055
**s350nm**	s350nm_l6000m_1	484.6	313.2	275.0	4.30	3.57	2.54	6.85	5.64	4.71	0.212	0.120	0.093	0.129	0.025	0.015
	s350nm_l6000m_2	622.5	537.3	344.5	5.84	4.46	2.80	9.16	7.38	5.15	0.184	0.223	0.104	0.068	0.102	0.045
	s350nm_l6000m_3	303.2	235.2	169.9	2.94	1.79	1.62	5.11	3.62	2.97	0.254	0.161	0.147	0.169	0.106	0.109
	s350nm_l6000m_4	351.4	325.1	339.3	3.36	2.78	2.82	5.78	4.90	5.18	0.130	0.180	0.131	0.053	0.051	0.013
	s350nm_l6000m_5	361.5	395.0	310.5	3.35	3.40	3.16	5.79	6.01	5.38	0.123	0.220	0.153	0.059	0.106	0.077
	s350nm_l6000m_6	315.5	462.3	304.6	3.12	4.23	2.75	5.56	7.28	4.85	0.126	0.172	0.135	0.072	0.087	0.094
**s400nm**	s400nm_l8000nm_1	391.8	564.2	449.8	3.81	5.29	4.49	6.74	8.89	7.41	0.131	0.179	0.136	0.035	0.087	0.059
	s400nm_l8000nm_2	361.2	576.3	351.4	3.69	5.17	3.53	6.26	8.48	5.94	0.140	0.172	0.128	0.084	0.063	0.062
	s400nm_l8000nm_3	363.7	390.8	265.1	3.91	4.11	2.67	6.57	6.34	4.74	0.159	0.180	0.111	0.089	0.076	0.069
	s400nm_l8000nm_4	281.1	479.5	314.0	2.98	4.17	2.65	5.07	6.86	4.87	0.171	0.192	0.143	0.091	0.077	0.052
	s400nm_l8000nm_5	457.8	500.0	365.3	4.14	4.69	3.29	6.96	7.92	6.12	0.147	0.190	0.147	0.040	0.083	0.085
	s400nm_l8000nm_6	345.1	506.7	318.4	3.48	4.28	3.55	5.93	7.55	5.72	0.155	0.164	0.136	0.061	0.072	0.026

For each sample ID, six 3D reconstruction images were generated from different regions of the same nanoporous gold (NPG) sample [8]. For each sample listed in Table 2, the corresponding 3D tomographic image, volume mesh file, engineering stress–strain and Poisson’s ratio data have been archived in *.tif, .inp, .txt* and *.txt* formats, respectively. Each file was named according to the sample name.

For example, for the sample *s20nm_l600nm_1*, the archived files are as follows:

1. **3D tomographic image**: *s20nm_l600nm_1.tif*
2. **Volume mesh file**: *s20nm_l600nm_1.inp*
3. **Stress–strain data**: *s20nm_l600nm_1.stress_strain.txt*
4. **Poisson’s ratio data**: *s20nm_l600nm_1.poisson_ratio.txt*

**Fig. 2:**
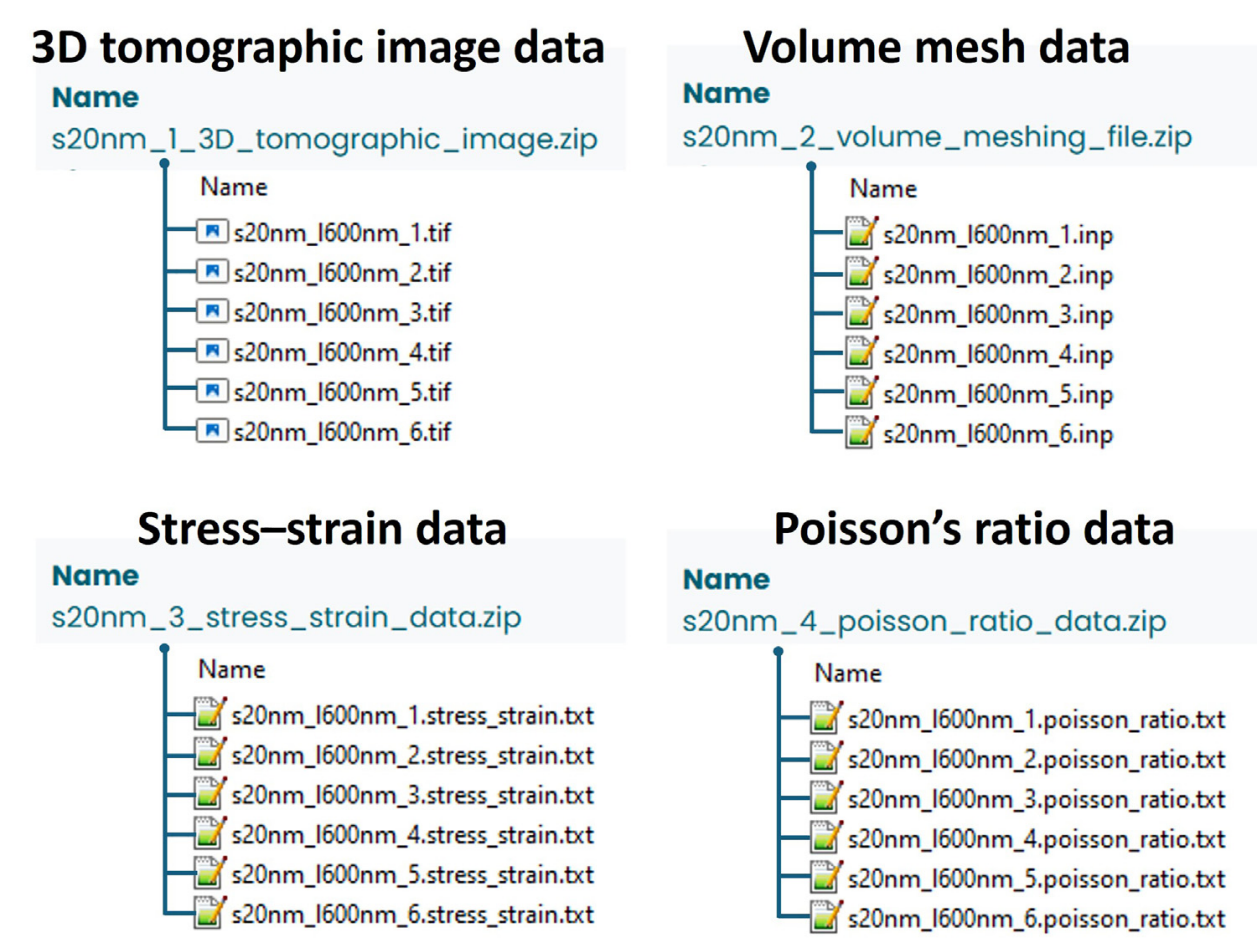
Structure of dataset files. For each sample ID, six 3D reconstruction images were generated from different regions of the same nanoporous gold (NPG) sample. Each sample contains four types of data: (1) 3D tomographic image data, (2) volume mesh data, (3) stress–strain data, and (4) Poisson’s ratio data. The example shown here corresponds to sample s20nm.

In the sample name *s20nm_l600nm_1*: “*s”* stands for ``sample'', “*20nm*” indicates a ligament size of ∼20 nm, “*l600nm*” denotes the sample edge length of 600 nm, and “*1*” refers to the first 3D reconstruction region within this NPG sample. The organization of the dataset files is illustrated in Fig. 2, and a README file describing the file structure has been uploaded to the repository.

The 3D topographies are stack tiff format image data which include slices in x−y plane that are stacked inz direction. The stack tiff file can be directly inputted into software like Fiji and Simpleware for further analysis or visualization. The volume mesh file generated by the software Simpleware is the input file for Abaqus. In the volume mesh file, the coordinates of each element, elements ID of each node were listed. The stress–strain data text file contains engineering stress–strain data for each sample under uniaxial compressive loading along the x, y, z directions. The sample edge lengths in each direction during compression, the engineering strain along the loading direction, and the Poisson’s ratios were analyzed and recorded in the Poisson’s ratio data files.

## 4 Experimental Design, Materials and Methods

### 4.1 3D tomographic reconstruction via FIB/SEM

Details of the nanoporous gold (NPG) sample preparation and 3D tomographic reconstruction methods can be found in Ref. [1]. Briefly, millimeter-sized NPG samples with a solid fraction of ∼0.30 were fabricated via electrochemical dealloying of Ag₇₅Au₂₅ solid solution. The as-dealloyed samples exhibited a ligament size of roughly 20 nm. To obtain samples with larger ligament sizes, the as-dealloyed NPG was annealed at 300 °C in air for varying durations. Prior to FIB/SEM reconstruction, the NPG samples were infiltrated with epoxy resin to facilitate focused ion beam (FIB) cross-sectioning and to enhance the contrast between gold ligaments and epoxy during scanning electron microscopy (SEM) imaging. The 3D tomographic reconstruction was carried out by alternating FIB milling and SEM imaging. The voxel size used during reconstruction ranged from 3.6 nm × 3.6 nm × 3 nm to 25 nm × 25 nm × 20 nm, depending on the ligament size of the NPG samples (Table 1). The 3D tomographic reconstruction data were saved in *.tif* format (e.g., *s20nm_l600nm_1.tif*) and archived in the “1_3D_tomographic_image” directory.

### 4.2 Volume meshing via Simpleware

Volume mesh files were generated using the segmentation software Simpleware (Synopsys, Inc. version Q-2020.06–1) [9]. Prior to import into Simpleware, isolated clusters, i.e. the disconnected components that do not contribute to the mechanical properties, were removed from the tomographic NPG structures using the “Cluster analysis” modifier in Ovito (version 3.0.0) [10] with the “*cutoff distance*” set to include only first nearest neighbors and non-periodic boundary conditions applied in all directions. The solid fraction before the removal of isolated clusters, $\varphi_{0}$, is calculated as the ratio of the number of solid voxels to the total number of voxels in the initial structure and is listed in Table 2. To import the structure into Ovito, the TIFF image file must first be converted into a compatible format, such as a LAMMPS dump file. After removing the isolated clusters, the data was converted back into a stack TIFF format. The preprocessed stack TIFF file was then imported into Simpleware. A new mask was created, and the images were binarized using the “Threshold” tool (lower value:128, upper value: 255). Finite element (FE) meshes were generated using the following settings:

• Mesh creation algorithm: FE Free
• Element type: All tetrahedra (quadratic, straight edges)
• Coarseness: -50

**Fig. 3:**
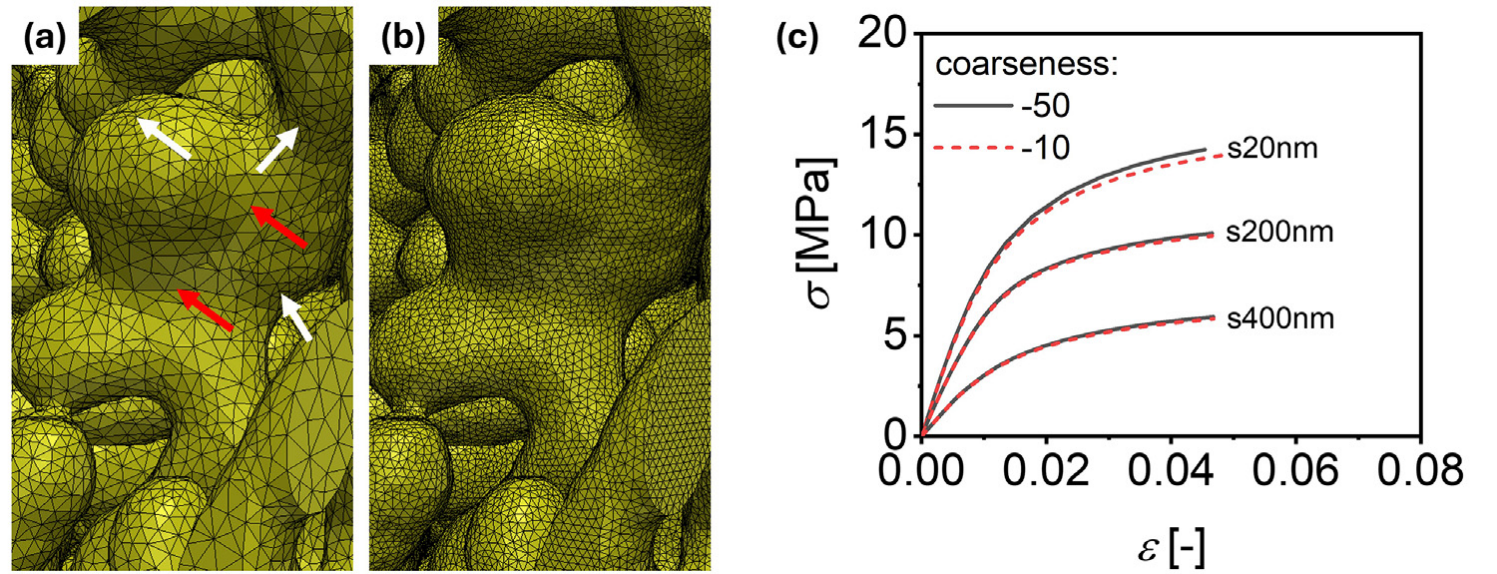
Effect of mesh Coarseness on volume segmentation and mechanical response. **(a)** and **(b)** show segmented ligament structures generated with mesh coarseness values of –50 and –10, respectively. The resulting finite element meshes contain approximately $1.32\;\times 10^{6}$ and $6.15\;\times 10^{6}$ quadratic tetrahedral elements (C3D10). Note the finer segmentation in the curved (white arrows) than flat (red arrows) regions of ligament and the much finer elements in (b). **(c)** Engineering stress–strain curves corresponding to both mesh resolutions for NPG samples with various ligament sizes show that the used values of mesh coarseness have a negligible effect on the mechanical response of the NPG structure. In (c), solid lines and short-dash lines represent “Coarseness” values of –50 and –10, respectively. Results are shown for three samples with different ligament sizes: s20nm, s200nm, and s400nm.

This “Coarseness: -50” was found to be sufficient for segmentation. Benchmark simulations comparing coarseness values of –50 and –10 for the same sample produced meshes with $1.32\;\times 10^{6}$ (Fig. 3a) and $6.15\;\times 10^{6}$ (Fig. 3b) elements, respectively. The engineering stress–strain curves obtained from FE simulations of samples with different ligament sizes, using the two coarseness values for segmentation, are shown in Fig. 3c. The differences between the results for the two coarseness values are small (relative error < 2 %), and even nearly identical in the elastic regime (strain < 0.002), validating the use of the “Coarseness: -50” parameter for segmentation.

The boundaries in the minimum and maximum of x, y and z directions were added to node sets for the boundary conditions setting up in Abaqus. The final volume mesh was exported as an *.inp* file (e.g., *s20nm_l600nm_1.inp*) and stored in the “2_volume_meshing_file” directory.

Structural properties including surface area, S, solid volume, $V_{s}$, and solid volume fraction, $\varphi_{m}$, were analysed through the statistic tools “Surface area”, “Volume” and “Volume fraction” in the “Measurements” of Simpleware respectively. The specific (per solid volume, $V_{s}$) surface area, $S_{V}$, was then calculated as $S_{V}=S/V_{s}$.

### 4.3 Finite element simulation in Abaqus

**Fig. 4:**
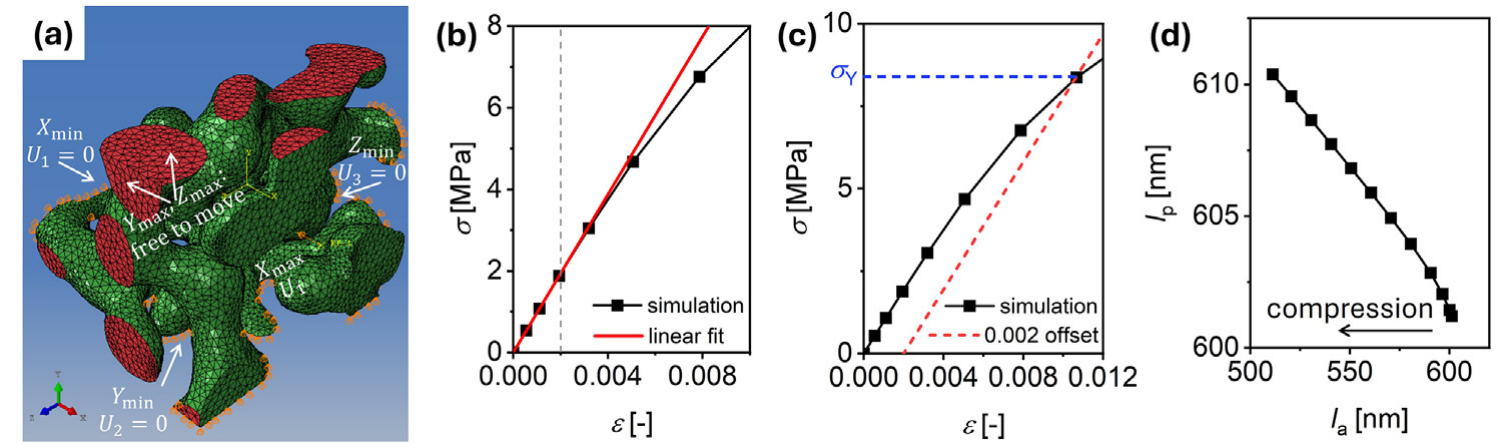
Schematic illustration of the boundary conditions in Abaqus and mechanical properties determination processes. **(a)** Schematic representation of the boundary conditions applied to the model in Abaqus, shown here for uniaxial compressive loading in the x-direction. The schematic depicts a subset region extracted from the full NPG sample. Fixed displacements $U_{1}=0$, $U_{2}=0$ and $U_{3}=0\;$boundary conditions were applied to the node sets at $X_{\text{min}}$, $Y_{\text{min}}$ and $Z_{\text{min}}$ respectively. A prescribed displacement, $U_{1}$was applied at the node set to be under compressive loading (“$X_{\text{max}}$” in this schematic), while node sets $Y_{\text{max}}$ and $Z_{\text{max}}$ were left free to move. **(b)** and **(c)** show the processes used to determine the Young’s modulus (E) and yield stress ($\sigma_{Y}$), respectively, from the engineering stress–strain curve. In **(b)**, E was obtained as the slope of a linear fit to the initial portion of the curve up to a strain of 0.002. In **(c)**, $\sigma_{Y}$ was identified as the intersection point between the stress–strain curve and a line offset by 0.002 strain from the initial linear fit. Another set of $\sigma_{Y}$ was also identified using a strain offset of 0.02. **(d)** The evolution of the sample edge lengths during compression in the direction of the applied displacement ($l_{a}$) and perpendicular to it ($l_{p}$).

Finite element (FE) simulations were performed using Abaqus (Dassault Systems Simulia Corp. version 2023) [11]. Fig. 4a illustrates the boundary conditions applied to the model. Symmetry boundary conditions were imposed on the node sets located at the minimum x, y and z boundaries. Uniaxial compression was simulated by applying a Displacement/Rotation boundary condition to the node set at the maximum boundary in the loading direction, while allowing free displacement in the other two directions. A previous study investigated FE simulations of NPG structures under both linear and non-linear geometry (see Fig. 3.15 in Ref. [12]). The non-linear geometry failed to converge at large strains and required significantly more CPU time. The small deviation between the two solutions with and without NLGEOM suggests that the impact of NLGEOM=NO is small also for larger strains [12]. In the present study, the FE simulations were therefore performed using a geometrically linear (NLgeom=off). The FE simulations required approximately 50, 40, 180, 480, and 600 CPU hours for the s20nm, s50nm, s200nm, s350nm, and s400nm samples, respectively.

Material properties used for gold in the simulation were as follows: Young’s modulus, 81GPa, yield stress, 700MPa, and Poisson’s ratio, 0.42. A work hardening rate of 1000 MPa was used, as suggested in Ref. [2]. The compression test was simulated up to a strain of 0.15.

Fig. 4b shows an example of the engineering stress–strain (σ−ε) curve from FE simulations. The Young’s modulus, E of the NPG samples was determined by fitting a straight line to the initial linear regime (up to strain of 0.002) of the stress–strain curve. The yield stress, $\sigma_{Y}$ was identified using the offset method at a plastic strain of 0.2 % (Fig. 4c): the intersection of the offset line with the stress–strain curve (indicated by the cross point in Fig. 4c) gives the yield point. Due to the early yielding of nanoporous gold at very small strains during compression [13], it is desirable to determine $\sigma_{Y}$ of NPG at a higher plastic strain to obtain a more stable value. The determination of yield stress at 2 % plastic strain follows the same procedure, but with an offset strain of 2 %. The engineering stress–strain data for each sample under uniaxial compressive loading in the x, y and z directions were archived in the “3_stress_strain_data” directory (e.g., *s20nm_l600nm_1.stress_strain.txt*).

Fig. 4d shows the evolution of the sample edge lengths during compression, both in the direction of the applied displacement ($l_{a}$) and perpendicular to it ($l_{p}$). The resulting $l_{p}-l_{a}$ relationship is nonlinear. The Poisson’s ratio ν was calculated using the following expression:

(1) $$\nu =-\frac{\delta \varepsilon_{p}}{\delta \varepsilon_{a}},$$

where $\delta \varepsilon_{a}$ and $\delta \varepsilon_{p}$ are the increments of true strain in the applied displacement and perpendicular directions, respectively. The true strain increment at time twas calculated as:

(2) $$\delta \varepsilon =\frac{l_{t}-l_{t-1}}{l_{t-1}},\;$$

where $l_{t}$ is the edge length of the sample at time t. The Poisson’s ratio was obtained by averaging the values calculated in the two transverse directions. The elastic Poisson’s ratio, $\nu_{E}$ was determined at a strain of 0.1 %, and the plastic Poisson’s ratio, $\nu_{P}$ at different engineering strain was evaluated within the plastic deformation regime (strain at 0.002–0.15). The sample edge length in each direction during compression, the engineering strain along the applied direction, the Poisson's ratios for the two perpendicular directions, and the averaged (with two perpendicular directions) Poisson's ratio were archived in the “4_poisson_ratio_data” directory (e.g., *s20nm_l600nm_1.poisson_ratio.txt*). The values of $\nu_{P}$ at a strain of 0.15 were listed in Table 2, while the $\nu_{P}$ values at other strains were archived in the “*.poisson_ratio.txt*“ files.

### 4.4 Topological connectivity via CHomP

**Fig. 5:**
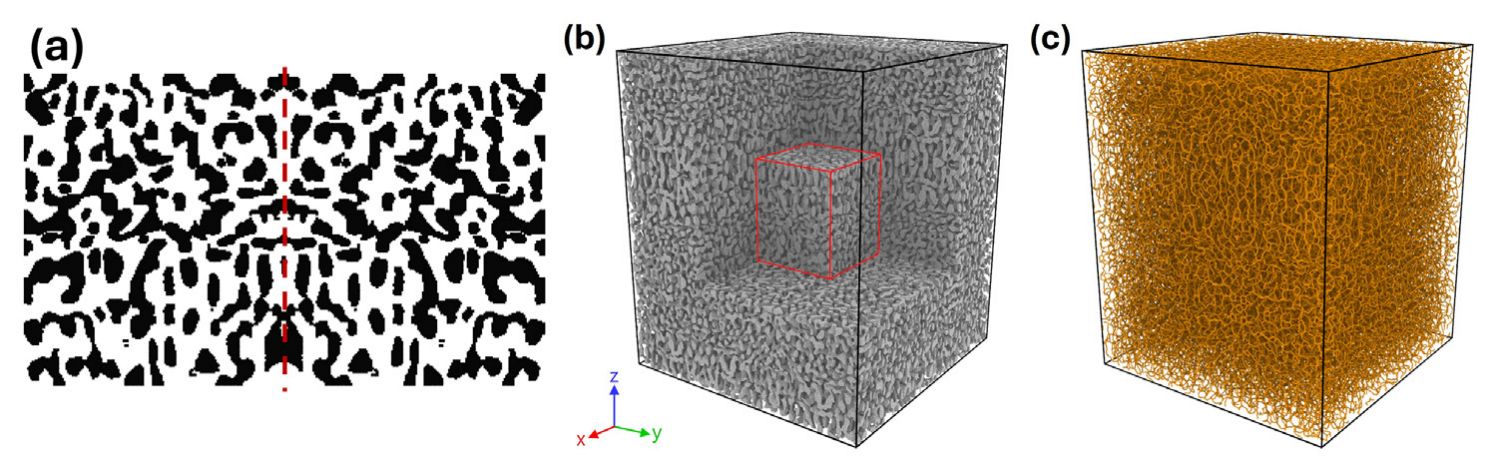
Schematic of mirror symmetry replication and skeletonization for genus measurements. **(a)** A 2D schematic demonstrating the mirror symmetry replication process. The original structure was mirrored along its edge (indicated by the red dashed line) to reduce boundary effects. **(b)** The extended structure, formed by surrounding the original RV (highlighted in red) with 26 mirrored replicas. Some surrounding RVs have been omitted for clarity to better visualize the initial RV (the central RV in red border). **(c)** The skeleton of the extended structure, generated using the “Skeletonise” function in the BoneJ plugin within FIJI [14], used for topological analysis via genus calculation.

To minimize the influence of non-periodic boundaries in the sample on the connectivity analysis, the 3D reconstructed structure was mirror-replicated along its faces and edges. Fig. 5a presents a 2D schematic where the original image (left) is symmetrically mirrored along its edge (indicated by the red dashed line). This approach was applied to three dimensions to produce an extended structure with a volume 27 times of the original one (Fig. 5b).

The skeleton of the extended structure, shown in Fig. 5c, was extracted using the “Skeletonise” function available in the BoneJ plugin for FIJI (version 6.5) [14]. This skeleton was then used to compute the genus, denoted as $G_{\text{extend}}$, which quantifies the number of connections of the NPG structure. The genus, $G_{\text{extend}}$, was computed using the open-source software CHomP (version 1.00) [15]. In CHomP, the Betti number, $B_{1}$—a topological invariant that quantifies the number of loops or ``handles'' in the structure—was calculated and used as a measure of the genus, providing a descriptor of the microstructure’s connectivity. A detailed description of the genus calculation using CHomP can be found in Ref. [3]. Non-periodic boundary conditions were applied in CHomP during the genus analysis.

The genus density, $G_{V}$, was calculated as:

(3) $$G_{V}=\frac{G_{\text{extend}}}{V_{\text{extend}}},$$

where $V_{\text{extend}}$ is the volume of the extended structure. The value of $G_{V}$ represents the genus density of the original structure.

The scaled genus, g, was calculated using the following expressions:

(4) $$g_{L}=G_{V}*L^{3}$$

and

(5) $$g_{\tilde{L}}=G_{V}*{\tilde{L}}^{3},$$

where scaling was performed using the ligament diameter L and the ligament spacing $\tilde{L}$, respectively.

The ligament diameter L was derived from the specific surface area $S_{V}$, using:

(6) $$L=\frac{4}{S_{V}}.\;$$

The ligament spacing $\tilde{L}$, defined as the distance between the centers of adjacent ligaments, was calculated from the dominant scattering vector $q_{0}$ of the small-angle interference function *S*(*q*) as:

(7) $$\tilde{L}=1.23\frac{2\pi }{q_{0}}\;.$$

**Fig. 6:**
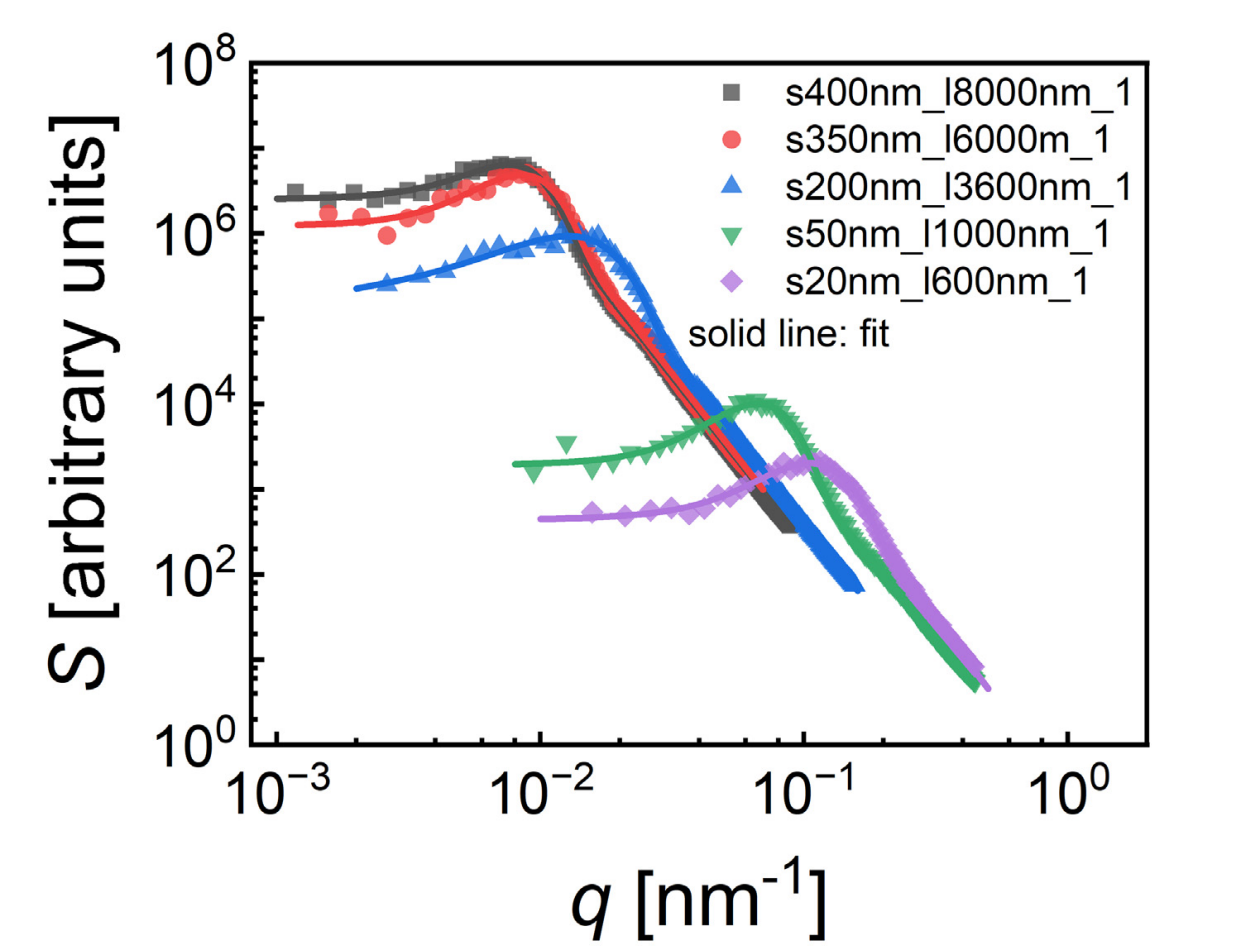
Small-angle interference function *S*(*q*) of nanoporous gold microstructures. Symbols represent the computed *S*(*q*) via a fast Fourier transform (FFT), as detailed in Ref. [16]. The solid line shows the corresponding fit using a Gaussian peak centered at $q_{0}$ combined with a baseline function of the form $A/{\left[1+Bq^{C}\right]}^{D}$ [17].

The interference function *S*(*q*) for each structure was obtained by applying a fast Fourier transform (FFT) to the 3D nanoporous gold structure from FIB/SEM reconstruction using the open-source software FFTW (version 3.3.8). The procedure for generating *S*(*q*) using FFT is described in detail in Ref. [16]. To extract $q_{0}$, the *S*(*q*) data was fitted using a combination of a Gaussian peak centered at $q_{0}$ and a baseline function of the form $A/{\left[1+Bq^{C}\right]}^{D}$ [17]. Fig. 6 shows the plot of *S*(*q*) versus q for samples with varying ligament sizes. The solid line represent the corresponding fitted curve, from which $q_{0}$ was extracted and subsequently used in Eq. (7) to compute $\tilde{L}$.

The value of $G_{V}$, the two types of ligament size (L and $\tilde{L}$), and the corresponding scaled genus ($g_{L}$ and $g_{\tilde{L}}$) for each NPG sample were listed in Table 2.

## Limitations

There are some limitations associated with both the FIB/SEM tomograph experiments and the FE simulations. One arises from the resolution of FIB/SEM tomographs for NPG samples with different ligament sizes. In coarsened structures with larger ligaments, each ligament cross-section is represented by more voxels compared to structures with smaller ligaments. A simple approximation can be obtained from the ratio of the mean ligament size to the voxel size shown in Table 1. For example, this ratio is 23.8 for the s400nm sample but only 7.3 for the s20nm sample. The slice thickness during 3D reconstruction [18], especially slicing lower than 10 nm applied to samples with small ligament sizes [19], may introduce uncertainty. Another limitation stems from the 3D reconstructed structures, which are non-periodic at their boundaries. As a result, only symmetric boundary conditions can be applied during FE simulations. This restriction excludes more complex loading configurations including shear components. Also, the FE simulations presented here do not account for surface effects, which are known to be size-dependent for nanoporous gold at the nanoscale.

## Ethics Statement

The authors have read and follow the ethical requirements for publication in Data in Brief and confirm that the current work does not involve human subjects, animal experiments, or any data collected from social media platforms.

## CRediT Authorship Contribution Statement

**Yong Li:** Simulation data curation, Formal analysis, Investigation, Writing – original draft. **Kaixiong Hu:** Experimental data curation, Formal analysis, Investigation, Writing – reviewing & editing. **Erica T. Lilleodden:** Funding acquisition, Project administration, Validation, Writing – reviewing & editing. **Norbert Huber:** Funding acquisition, Project administration, Validation, Writing – reviewing & editing.

## Data Availability

TOREDatasets for structural and mechanical properties of nanoporous networks from FIB reconstruction (Original data).
